# Impact of risk perception on emergency information seeking behavior: a meta-analysis

**DOI:** 10.3389/fpsyg.2025.1646584

**Published:** 2026-01-09

**Authors:** Wanlan Gong, Zhihong Li, Jun Tang

**Affiliations:** School of Public Administration and Policy, Renmin University of China, Beijing, China

**Keywords:** disaster type, information seeking behavior, meta-analysis, risk perception, theoretical framework

## Abstract

**Purpose:**

Risk perception significantly impacts how individuals assess risk, make decisions, and behave. While numerous studies have examined risk perception's impact on emergency information seeking behavior, the nature of the association remains unclear.

**Methods:**

This study established a theoretical framework, and a meta-analysis was conducted to examine risk perception's impact on emergency information seeking behavior. Fifty relevant studies (29,014 participants) covering risk perception and information seeking behavior data in four emergency scenarios were included.

**Results:**

A significant positive correlation was found between risk perception and emergency information seeking behavior. Further exploratory analysis indicated different impacts of risk perception on information seeking behavior in each type of emergency (natural disasters, public health accidents, and social security emergencies). Health and natural disaster emergencies had a significant positive moderating effect, whereas accidents and social security emergencies had a significant negative moderating effect. We found significant differences in the moderating effects of demographics (national development level and male proportion) and methodology (i.e., publication time, sample collection strategy, and measurement method). Furthermore, we evaluated the publication bias and literature quality to determine the robustness and scalability of the results.

**Conclusion:**

To the best of our knowledge, we present the first meta-analysis study on risk perception and emergency information seeking behavior, summarizing the rich empirical knowledge on these relationships. This study followed contemporary meta-analysis guidelines and best practices to generate transparent and replicable scientific findings. Our findings can help improve information dissemination's effectiveness in emergency situations and offer a theoretical foundation for strengthening public emergency response capabilities.

## Introduction

Emergency situations are constantly evolving; hence, understanding individuals' emergency information-seeking behavior is important. In recent years, the world has experienced various emergencies threatening public safety and social stability. For example, the average annual occurrence of natural disasters (e.g., earthquakes, floods, and typhoons) increased by 40% compared to the previous decade (David et al., 2024; UNDRR, 2022; Piracha, 2021). Public health emergencies, such as new infectious disease outbreaks and food safety crises, are growing rapidly each year (Li and Li, 2024; Park et al., 2024; Zhao and Cai, 2009), while industrial accidents and traffic disasters result in approximately 2.7 million casualties annually (David et al., 2024; Fung, 2024); moreover, social security issues, such as terrorist attacks and mass incidents, persist. These events have affected over 90% of countries in the past 5 years, with an average annual growth rate of over 2.5% (Chao et al., 2016; Kaur, 2018; OECD, 2025). The high frequency and complex characteristics of these emergency events not only directly impact life and property but also pose serious challenges to the information processing and behavioral decision-making mechanisms of individuals in crisis. Therefore, this topic attracts widespread attention from scholars in disaster management, public health, psychology, and other fields, as well as policymakers and emergency managers.

When emergencies such as natural disasters, public health events, industrial accidents, and social security issues occur, people's ability to quickly and effectively gather information significantly affects the outcomes of emergency responses and the extent of losses (Carson et al., 2023; Huang et al., 2023; Kellens et al., 2012). Emergency information-seeking behavior refers to the actions taken by individuals to actively obtain information related to emergency situations, including the nature of the emergency, potential risks, response strategies, and rescue measures (Carson et al., 2023; Kellens et al., 2012). This information-seeking process is typically driven by an individual's risk perception. Risk perception is an individual's subjective assessment of the potential threat posed by an emergency situation (Li et al., 2020; Ellen and Jan, 2008; Slovic, 1987). Risk perception affects how individuals assess risk, make decisions, and behave. Several factors influence risk perception, including the emergency situation's characteristics, personal experiences, and social and cultural environments (Kellens et al., 2012; Slovic, 1987; Li et al., 2020, 2024). In emergency situations, people with higher risk perception tend to be more anxious and uncertain, which motivates them to seek relevant information to reduce their uncertainty and help them make informed decisions (Kim and Madison, 2020; Slovic, 1987). For example, during the sudden COVID-19 outbreak, people who perceived a higher infection risk were more likely to actively search for information on disease prevention, treatment, and control measures. When people perceive a lower risk of sudden health emergencies or other crises, they do not actively seek accurate information, which may exacerbate the risk (Shen et al., 2022; Zhang et al., 2022).

Over the past few decades, numerous studies have investigated the relationship between risk perception and emergency information-seeking behavior; however, the results have been inconsistent. Some studies reported a positive correlation between risk perception and emergency information seeking behavior (e.g., Carson et al., 2023; Hovick et al., 2014; Kahlor, 2010; Kellens et al., 2012; Li and Li, 2024; Liao et al., 2018; Park et al., 2024; Wen, 2019; Zhang et al., 2022; Zhao and Cai, 2009), whereas others found either a negative (e.g., Chao et al., 2016; David et al., 2024; Kim and Madison, 2020; Mou et al., 2016; Real, 2008) or no correlation between the two (e.g., Broermann, 2018; Li et al., 2017; Liu et al., 2021; Wang et al., 2016; Zhang, 2017). These inconsistencies can be attributed to differences in the theoretical perspectives, research backgrounds, sample characteristics, and research methods used. For example, studies conducted in different types of emergency situations (e.g., natural disasters and public health events) may yield different results (Kahlor, 2010; Kellens et al., 2012). Additionally, individual differences—such as age, gender, education level, and personality traits, as well as changes in information dissemination channels (such as traditional and social media)—may also affect this relationship (Li et al., 2020, 2024). To date, only two studies have reviewed the literature on risk perception and emergency information seeking behavior (Anthonj et al., 2022; Fu et al., 2025). However, these were qualitative summaries. For example, Anthonj et al. (2022) qualitatively summarized whether perceived potential risks to health and happiness affect health promotion and/or health-seeking behavior, whereas Fu et al. (2025) qualitatively outlined how factors such as the digital divide, social media influence, public health initiatives, risk perception, and health anxiety affect online health information seeking behavior; these approaches may foster a subjective distortion of empirical data (Hedges and Tipton, 2009). By contrast, a meta-analysis provides a highly systematic, objective, transparent, and reproducible method for reviewing empirical literature. It outlines strategies for collecting primary research, applying selection criteria, developing and cross-validating variable categories, and synthesizing research results. A meta-analysis has high statistical power and can detect small effects by combining research results, which can reveal the diversity of research results and explain differences between studies (Barends and Rousseau, 2018; Gurevitch et al., 2018; Li et al., 2020; Tang and Li, 2025).

**Figure 1:**
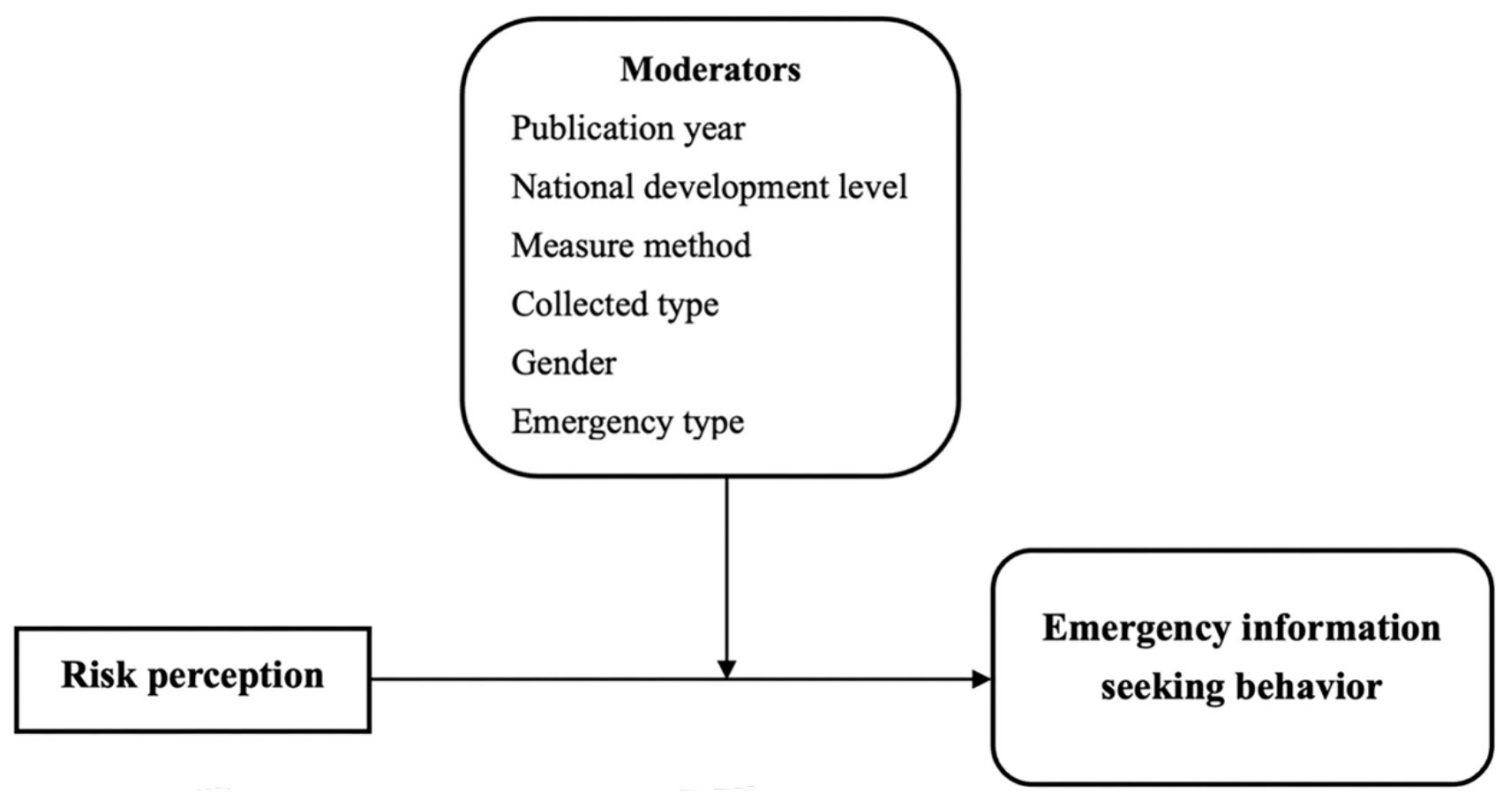
The study's theorical framework.

Considering the inconsistent conclusions regarding the relationship between risk perception and emergency information-seeking behavior, this systematic review aimed to provide clarity by reviewing 50 studies to determine the nature of this relationship. Figure 1 illustrates this study's theoretical framework, which was used to address the following three questions:

Q1. What is the relationship between risk perception and emergency information seeking behavior?

Q2. What potential factors explain the differences between studies?

Q3. What is the significance of the future emergency management research and policy implementation?

## Theoretical background

### Risk perception

Risk perception is an individual's subjective perception and risk evaluation, including the likelihood of risk occurrence, potential losses, and the importance of these losses to the individual (Slovic, 1987). In the emergency management field, risk perception refers to an individual's perception and assessment of various potential hazards (such as natural disasters, public health events, etc.), which affects their level of concern regarding risks and their motivation to implement response measures (Kellens et al., 2012; Li et al., 2020, 2024). For example, in the face of an epidemic, individuals' perception of infection risk affects their willingness and effort to obtain relevant information.

Risk perception includes dimensions such as the likelihood of risk occurrence, severity of risk consequences, and an individual's controllability of risk. These dimensions are interrelated and constitute an individual's overall risk perception. For example, individuals may believe that the likelihood of an earthquake occurring in a certain location is low; however, if an earthquake occurs, the consequences are serious, which can also affect individuals' overall perception of earthquake risk (Li et al., 2020).

Various factors—including an individual's personal experience, knowledge level, and sociocultural background—influence risk perception. Additionally, risk perception dynamically adjusts to changes in time and context. For example, individuals who have experienced earthquakes may have a deeper understanding of earthquake risks, fostering greater alertness and positivity toward subsequent emergency preparedness. Moreover, external information—such as media reports and expert advice—can affect individuals' risk perception (Li et al., 2020; Slovic, 1987).

The core theoretical framework of risk perception involves individuals' subjective cognition of the possibility, severity, and controllability of risk events (Slovic, 1987). Its theoretical development is primarily based on Protection Motivation Theory (PMT) and the Theory of Planned Behavior (TPB). Notably, PMT posits that individuals develop protective motivation upon perceiving a threat, which in turn drives adaptive behaviors, such as information seeking (Rogers, 1983). Further, TPB emphasizes the influence of subjective norms and perceived behavioral control on behavioral intention, and risk perception—as an attitude variable—indirectly affects information seeking behavior through behavioral intention (Ajzen, 1991).

### Emergency information seeking behavior

Emergency information-seeking behavior refers to individuals actively seeking information associated with risks when facing emergency situations, including information on the nature of the emergency, possible impacts, and response measures (Carson et al., 2023; Kellens et al., 2012). This behavior is intended to help individuals better understand risks, reduce uncertainty, and make wiser decisions to protect their own safety and that of others.

Emergency information seeking behavior is purposeful and proactive. After perceiving risks, individuals selectively search for and obtain relevant information based on their needs and goals. Simultaneously, various psychological factors—such as individual cognition, emotion, and motivation—influence this behavior. For example, in the early stages of a viral outbreak, people may actively search for information regarding the transmission routes, symptoms, and prevention methods of the virus to satisfy their need to understand and respond to the epidemic (Carson et al., 2023; Kellens et al., 2012).

Information seeking in emergency situations is the process whereby individuals actively acquire information to reduce uncertainty and satisfy their decision-making needs (Kellens et al., 2012). Information Needs Theory suggests that when risk perception exceeds a threshold, individuals' subjective need for information increases, prompting them to invest more resources in information searches. Cost-Benefit Theory suggests that information seeking behavior is the result of individuals balancing time cost, cognitive load, and information value. High risk perceptions may enhance individuals' evaluations of information value, thereby increasing their willingness to search (Carson et al., 2023; Kellens et al., 2012). These theories suggest potential mechanisms driving emergency seeking behavior.

### Theoretical perspective

Each theoretical framework focuses on different interpretations of the risk–behavior relationship. Notably, PMT emphasizes the dual pathway regulation of threat assessment and response effectiveness (Rogers, 1983), which may overestimate the mediating role of cognitive assessment, while the Risk Information Seeking and Processing Model (RISP) focuses on the interactive effects of emotional responses and information sufficiency (Griffin et al., 2008). A meta-analysis found that studies using the TPB were more likely to identify the moderating effects of subjective norms (with an average effect value reduction of 0.07–0.12), indicating that the choice of theory may systematically affect the interpretation of results.

### Relationship between risk perception and emergency information seeking behavior

Risk perception and emergency information-seeking behavior have a close relationship. The higher an individual's risk perception, the stronger their emergency information-seeking behavior (Kellens et al., 2012). This is because when individuals perceive higher risk, they feel greater pressure and uncertainty, which prompts them to actively seek relevant information to reduce this pressure and uncertainty to protect themselves. Additionally, emergency information-seeking behavior is a goal-directed behavior whereby individuals actively obtain the “essence of the event, scope of impact, and response measures” to reduce uncertainty and protect their own and others' safety (Kellens et al., 2012). The occurrence of this behavior requires simultaneous satisfaction of the “motivation drive” (risk perception triggering), “conditional support” (behavioral control ability), and “emotional regulation” (emotional and information sufficiency perception), which correspond to the core concerns of PMT, TPB, and RISP, respectively, and constitute the integrated framework's behavioral endpoint (Li et al., 2020; Tang and Li, 2025).

Numerous studies have demonstrated that risk perception is an important factor affecting emergency information-seeking behavior. For example, in the field of natural disasters, studies have found that the higher people's perception of earthquake risk, the greater their likelihood of actively obtaining earthquake warning information and emergency knowledge before an earthquake occurs (David et al., 2024; Piracha, 2021). During public health events, people's perceptions of the epidemic risk can also affect their attention to and search frequency for epidemic-related information (Li and Li, 2024; Park et al., 2024; Zhao and Cai, 2009). Some studies have suggested that this relationship may be moderated by various factors such as individual differences, situational factors, and information dissemination channels. For example, individuals of different ages and educational backgrounds may have different perceptions of risk and information-seeking behavior, and the relationship between risk perception and information-seeking behavior may vary across diverse sociocultural contexts (Li and Yang, 2025). Therefore, an in-depth exploration of the relationship between risk perception and emergency information seeking behavior, as well as the moderating factors that affect this relationship, is of great significance for improving emergency management and enhancing the public's emergency response capabilities.

### Moderators

#### Publication time

Publication time may moderate the relationship between risk perception and emergency information-seeking behavior, reflecting the evolving trends in the research field over time. One study found that because the information environment is dominated by traditional media, public risk perception is primarily influenced by one-way communication from authoritative institutions, and information seeking behavior relies more on passive reception (Anthonj et al., 2022). With the popularization of social media, the diversification of information acquisition channels may enhance risk perception's effect on active searches (Shen et al., 2022).

#### Cultural background

At the national development level, the public in developed countries may rely more on institutionalized information channels (such as government warning systems) owing to improved infrastructure and widespread risk education. The correlation between risk perception and information search exhibits a rational characteristic (Kellens et al., 2012; Mokry, 2019); However, developing countries have limited resource accessibility and rely more on interpersonal networks and informal channels for risk response, which may enhance the direct driving force of the perception of search behavior (Wen, 2019). In the regional cultural dimension, Asian regions characterized by collectivist learning are more concerned with sharing group risk information (Wen, 2019), while individualistic-dominated European and American regions place more emphasis on individualized information screening (Kellens et al., 2012), which may result in intercontinental differences in effect values.

#### Methodologies

Concerning measurement methods, structural equation modeling (SEM) can more accurately capture the multidimensional features of risk perception (such as cognitive/affective components) through latent variable modeling, and its effect values are generally 0.15–0.20 higher than those of multi-layer regression (Chen, 2018). Multi-layer regression may weaken the main effect's significance when controlling for nested individual data, such as family/community. Regarding data collection methods, online surveys cover high-risk and sensitive populations (such as young and highly educated groups), and their risk perception–information-seeking correlation is 0.08–0.12 higher than offline interviews (Wang et al., 2022). However, there may be social approval bias.

#### Emergency type

The intensity of disaster characteristics has a significant moderating effect. Natural disasters (such as earthquakes/floods) are more prone to active information seeking by the public due to their predictability and accumulated historical response experience (with an effect value of 0.51). However, information overload and contradictions in public health events, such as epidemics, may trigger “vigilance fatigue” and weaken the behavioral transformation of risk perception (the effect value decreases to 0.35) (Piracha, 2021; Siegrist and Zingg, 2014). Additionally, industrial accidents involve complex attributions of responsibility, and social security incidents involve political sensitivity, both of which may indirectly affect information-seeking paths through trust mechanisms (Chao et al., 2016; David et al., 2024; Kaur, 2018; Terpstra, 2011).

## Materials and methods

### Search strategy

**Figure 2:**
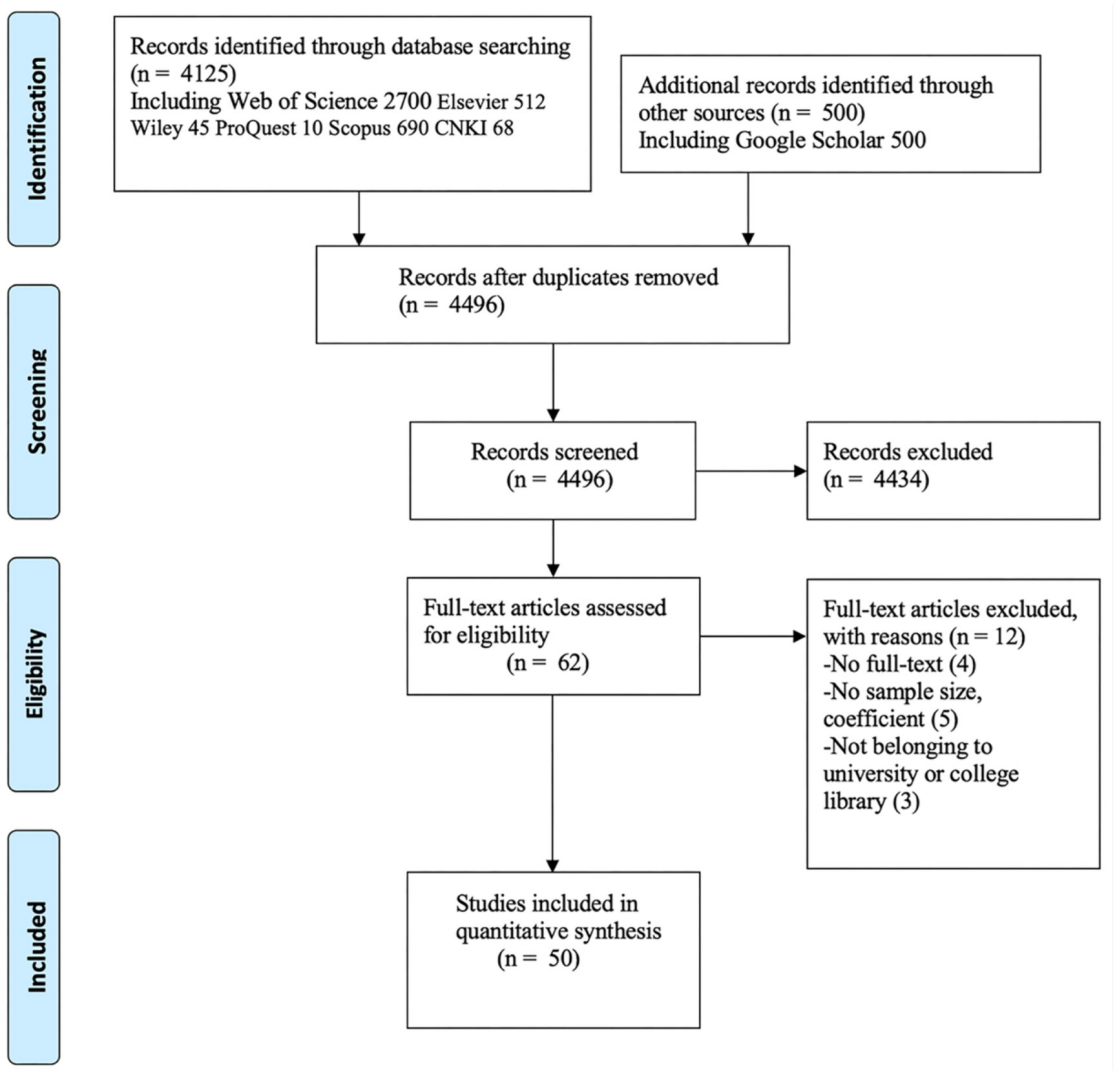
PRISMA diagram for search and selection process.

When conducting and reporting our system review, we followed the Preferred Reporting Items for Systematic Reviews and Meta-Analyses (PRISMA) guidelines (Moher et al., 2009) and conducted a systematic search with the participation of professional research librarians to identify relevant literature. Firstly, on May 2th, 2025, we searched the following these international electronic databases (Web of Science, Scopus, Elsevier, Wiley, ProQuest, CNKI, and Google Scholar) were searched to identify potentially relevant published and unpublished studies (Barends and Rousseau, 2018). We combined keywords and Boolean search strings (such as “^*^”, “NOT”, “OR”, and “AND”) for systematic search: for example, in the Web of Sciences database, we used TS=(“risk perception^*^” OR “perceived risk^*^” OR “risk awareness”) AND TS=(“emergency information^*^” OR “crisis^*^ information^*^” OR “disaster information^*^”) AND TS=( seek^*^ OR search^*^ OR retriev^*^ OR find^*^ OR use^*^) AND TS=(behavio^*^ OR intention^*^ OR willingness^*^) for retrieval, where TS is the subject word. Secondly, to minimize the impact of publication bias and obtain as the maximum number of potentially interesting studies as possible, we have also included unpublished conference papers, research proposals, and papers in the aforementioned database. Finally, we use the snowball method was used to review the reference sections of the literature obtained to identify other potential sources. A total of 632 studies were reviewed, and those that met the systematic review criteria were included. The search process is illustrated in Figure 2.

### Inclusion and exclusion criteria

The inclusion criteria for constructing the dataset were as follows: (a) this study included any types of research design articles that evaluate the risk perception and emergency information seeking intention or behavior; (b) the study was a quantitative study, with the outcome indicators being emergency information seeking intention or behavior; and (c) this study reported the correlation coefficients [or other convertible coefficients, such as d, r^2^, and standard error (SE)] and the sample size. We excluded studies that (a) did not investigate the risk perception and emergency information seeking intention or behavior; (b) duplicated results or studies; or (c) lacked full-text information sources such as comments, meeting minutes, or editorials.

### Coding and data extraction process

To verify the accuracy of the coding, a coding team was established comprising three researchers with health and management knowledge as well as methodological experience. The coding process was completed by two researchers who independently coded each included study. First, through frequent discussions between the coding teams, the two researchers reached an agreement on coding standards and began the first round of experimental coding, covering many features that the coding team believed might be related to post-hoc analyses. These include, but are not limited to, author, publication year, region, sample size, demographic characteristics (gender, age, and education level), risk perception, region, theoretical background, measure method, and the main findings. Second, the two researchers independently encoded all the relevant research features based on the revised coding scheme. Third, two researchers cross-checked and verified that the coder's coding consistency reached 85% and then independently screened the title and abstract to determine which studies could be included. Articles that could not be identified by their titles and abstracts were screened for full-text. When the two researchers had different opinions, a third researcher decided whether to include or exclude the study. The two researchers independently extracted the data from the included studies and merged the results into a final data table. During the encoding and extraction processes, differences and disagreements were resolved through discussion and/or by a third reviewer. All excluded articles and the reasons for exclusion were explained.

### Effect size calculation

Most included studies are based on reported correlation coefficients that examine the relationship between risk perception and information behavior. The correlation coefficient is usually marked as “R” or “r”. However, other coefficients [such as R^2^, d, or standard deviation (SD)] have also been used in some studies. In this case, it is necessary to convert these coefficients into r, as follows (Others can be find in Barends and Rousseau, 2018; Card, 2011; Li et al., 2020):


$$\begin{array}{l} d=\frac{m1-m2}{s}\\ r=\frac{d}{\sqrt{4+d}} \end{array}$$

where m1 and m2 are the means of Groups 1 and 2, respectively, and *s* is the pooled SD across the two groups.


$$\begin{array}{l} r=\frac{{\left(1-R^{2}\right)}^{2}}{n-1} \end{array}$$

*R*^2^ was the determinant coefficient, and *n* was the sample size.


$$\begin{array}{l} Z_{i}=0.5ln\left(\frac{1+r_{i}}{r_{i}}\right),SE_{i}=\frac{1}{\sqrt{W_{i}}},Z=\frac{\sum \left(W_{i}*Z_{i}\right)}{\sum W_{i}},r=\frac{e^{2z}-1}{e^{2z}+\;1} \end{array}$$

*W*_*i*_ = *n* – 3, where Z is the Z-score when calculating Fisher's Z.

### Risk-of-bias assessment

We conducted a bias risk assessment of the included emergency information seeking studies and the data were independently extracted by two researchers. Bias risk assessment is a measurement tool used to evaluate the quality of research and can provide a reference for the robustness and scalability of the research results. It comprises seven items, with the answer options of “yes” “no” and “unclear” for each item. This tool was adapted from previously developed bias risk tools for observational research (Bero et al., 2018; Higgins et al., 2024; Schünemann et al., 2013).

### Data analysis

To determine the relationship more truthfully and reliably, we used Schmidt and Hunter's random effects model (2015) for analysis; Microsoft's Excel 2021 (version 16.76) was used to extract and organize data; R (version 4.3.1)'s “metafor” package and Comprehensive Meta Analysis Version 3 software (CMA 3.3.07; Biostat, USA) were used for meta-analysis, moderator analysis, publication bias analysis, etc. First, we used the correlation coefficients reported in each study, the internal consistency reliability coefficient (α), and then we calculated the true correlation coefficient (p) between risk perception and emergency information seeking behavior (p also known as the zero-order correlation coefficient, which is the correlation coefficient after weighted observation of the average sample size or the true correlation coefficient after measurement error correction). For the few studies that reported other effect values (such as d, mean, and SD), we converted them into a unified comprehensive effect value r. For studies without reported reliability, internal consistency reliability tends to overestimate the reliability of score-based standards (and, conversely, underestimate the correlation of corrections), α corrected the magnitude of the attenuation effect, as internal measurement errors are usually smaller than inter measurement errors (Lebreton and Senter, 2008). Therefore, we used the weighted average p of all studies that reported the reliability of this variable. For studies that included multiple and/or outcome measurements, we calculated a comprehensive r for inclusion in the overall meta-analysis (Barends and Rousseau, 2018). Subsequently, to better evaluate the impact of publication bias in the included studies, we used a combination of the Failsafe N (Rosenthal) and Egger regression analysis methods, as both methods have advantages and disadvantages in testing different samples. For example, the Egger regression analysis is prone to errors when the sample size is small. Therefore, it is unsuitable for testing small-sample studies. Failsafe N (Rosenthal) can compensate for this deficiency, which reflects the number of studies required to reverse the results of the meta-analysis and estimate the impact of potential unpublished negative results on positive meta-analysis results (Barends and Rousseau, 2018; Li et al., 2020). When the number of Failsafe N was much greater than the number of included studies, particularly when the number of studies exceeded 5 K + 10, where K is the number of included studies (Card, 2011), it is difficult to change the results of the meta-analysis. However, its judgment criteria are not the golden rule; thus, we combined the two to evaluate the impact of publication bias on the included studies. This is conducive to reflecting the robustness of the meta-analysis results regarding any systematic omissions in the published literature that yielded no significant results. Finally, the quality of the included studies was evaluated as the basis for the reliability and scalability of the research results.

## Results

After searching the database and organizing the relevant articles, a large amount of valuable information was collected. Subsequently, we provide a basic description of this information.

### Study characteristics

After screening, 50 publications were included, with a total sample size of 29,014 participants, most of whom were female (50.69%). These studies involved nine countries across three continents, covering both developing and developed countries, and demonstrated significant growth trends over the past 5 years (2021–2025). Specifically, the samples came from the following continents and countries: Asia (k = 36), North America (k = 7), Europe (k = 5), and Africa (k = 2); China (k = 32), United States (k = 7), South Korea (k = 3), South Africa (k = 2), Netherlands (k = 2), Spain (k = 1), India (k = 1), Belgium (k = 1), and Greece (k = 1). The vast majority of research came from developing countries (k = 35). Among the 50 included studies, from a theoretical perspective, most were based on TPB and the theory of reasoned action (k = 27), while grounded theory, PMT, Uncertainty Theory, and others were relatively rare. The research methods mainly included SEM (k = 37) and hierarchical regression (HR) (k = 13). Online sample collection methods (k = 23) included email, online questionnaires, and telephone interviews; offline methods (k = 19) included distributing questionnaires and conducting onsite interviews; and mixed collection involved combining online and offline methods (k = 8). Among the four types of emergency situations, most were related to health (k = 26), social safety (k = 15), natural disasters (k = 6), and accident emergencies (k = 3). Table 1 presents detailed information.

**Table 1:** General characteristics of the 50 included studies.

**No**.	**Article ID**	**Publication year **	**Location **	**Locationcode **	**National development level **	**Main findings **	**Theoretical perspective **	**Journals **	**Measuremethod **	**Valid sample size **	**Population types **	**Colleted type **	**Gender (male%) **	**Emergency type **	**Risk perception **	**Emergency information seeking behavior **
1	Li and Li, 2024	2024	China	Asia	Developing	Health risk perception has a significant positive impact on health information seeking.	Health belief model and protection motivation theory	Behavioral Sciences	SEM	885	Youth	Online	53.79%	Health emergency	Risk perception	Behavior
2	Park et al., 2024	2024	Korea	Asia	Developed	Information-seeking behaviors affect infection-prevention behaviors directly and indirectly through risk perception, with trust in media and government moderating this relationship. Higher trust levels lead to consistent compliance with preventive behaviors regardless of risk perception.	Protection motivation theory	Patient Preference and Adherence	SEM	700	Adults	Online and offline	49.60%	Health emergency	Risk perception	Behavior
3	Zhang et al., 2022	2022	China	Asia	Developing	Health risk perception has a significant positive impact on health information search behavior.	Risk information search and processing model	Frontiers in Public Health	SEM	646	Older people	Online	49.60%	Health emergency	Risk perception	Behavior
4	Shen et al., 2022	2022	China	Asia	Developing	Specific types of channels of information acquisition and public trust in these information channels, their informational content, and proportion of negative information, as well as a frequency of information seeking all had an impact on risk perception during COVID-19.	Social amplification of risk framework	Psychology Research and Behavior Management	SEM	2611	Adults	Online	29.26%	Health emergency	Risk perception	Behavior
5	Mokry, 2019	2019	USA	North America	Developed	Risk perception has a positive impact on emergency information seeking intention and behavior.	Theory of planned behavior	Colorado State University	SEM	710	Farmers	Online	62.00%	Natural disaster emergency	Risk perception	Intention, Behavior
6	Wen, 2019	2019	China	Asia	Developing	Bridging and bonding social capital directly and indirectly predicted risk information seeking regarding genetically modified organisms (GMOs).	Risk information seeking and social capital models	Journal of Risk Research	SEM	1286	Citizens	Online	50.30%	Natural disaster emergency	Risk perception	Behavior
7	Deng and Liu, 2017	2017	China	Asia	Developing	Perceived health risk has a significantly negtive influence consumers' health information-seeking behavior intention.	Theory of reasoned action	International Journal of Medical Informatics	SEM	436	Patients with non-serious conditions	Online	52.50%	Health emergency	Risk perception	Intention
8	Mou et al., 2016	2016	South Africa	Africa	Developing	Perceived health risk has a significantly negtive influence health information-seeking behavior intention.	Theory of planned behavior	Information Technology and People	SEM	703	Young college students	Online	46.50%	Health emergency	Perceived performance risk	Intention
9	Kaur, 2018	2019	India	Asia	Developing	Investors having poor risk perception tend to reduce their bias by accessing personal sources of information.	Theory of planned behavior	Qualitative Research in Financial Markets	SEM	225	Mutual fund investors	Offline	91.10%	Social safety emergency	Risk perception	Behavior
10	Zhao and Cai, 2009	2009	Greece	Europe	Developed	Risk perception has a positive impact on health emergency information seeking behavior.	Theory of planned behavior	Health Communication	SEM	340	Lung cancer patients	Online	39.00%	Health emergency	Perceived personal risk	Behavior
11	Liao et al., 2018	2018	China	Asia	Developing	The results show that current knowledge, risk perception, perceived channel beliefs, and perceived information-gathering capacity (PIGC) are all significant predictors of information need and information-seeking intention.	Theory of planned behavior	International Journal of Environmental Research and Public Health	SEM	731	Residents	Online	46.00%	Health emergency	Risk perception	Intention
12	Li et al., 2017	2017	China	Asia	Developing	Risk perception has an indirect effect on earthquake risk information seeking behavior via information need.	Theory of planned behavior	International Journal of Environmental Research and Public Health	SEM	918	Residents	Offline	52.10%	Natural disaster emergency	Risk perception	Behavior
13	Kahlor, 2010	2010	USA	North America	Developed	Risk perception has a positive impact on emergency information seeking intention.	Theory of planned behavior	Health Communication	SEM	804	Residents	Online	40.00%	Health emergency	Risk perception	Intention
14	Hovick et al., 2014	2014	USA	North America	Developed	Risk perception has a positive impact on emergency information seeking intention.	Theory of planned behavior	Journal of Health Communication	SEM	1007	Adults	Online	38.00%	Health emergency	Risk perception	Intention
15	Chao et al., 2016	2016	China	Asia	Developing	All preliminary findings indicate that both beliefs and perceived risks of users in the search engine positively affects their behavioral intention.	Theory of planned behavior	Computers in Human Behavior	SEM	890	Network users	Online	49.00%	Social safety emergency	Privacy risk	Intention
16	Wang et al., 2016	2016	China	Asia	Developing	Risk perception has no impact on emergency information seeking behavior.	No reported	Chinese Journal of Management Science	HR	363	Adults	Online and offline	48.00%	Social safety emergency	Risk perception	Behavior
17	Hu, 2017	2017	China	Asia	Developing	The perceived risk of infant formula buyers has a negative impact on their search behavior, but the relationship is not significant.	Theory of planned behavior	Huazhong Agricultural University	SEM	212	Adults	Online and offline	36.20%	Social safety emergency	Risk perception	Behavior
18	Wang, 2018	2018	China	Asia	Developing	Perceived risk has a significant impact on WeChat consumer information search behavior.	Theory of planned behavior	Jiangsu University of Science and Technology	SEM	418	WeChat users	Offline	35.90%	Social safety emergency	Risk perception	Behavior
19	Zhang, 2017	2017	China	Asia	Developing	Risk perception has no impact on emergency information seeking behavior.	Theory of planned behavior	Guangxi University	HR	333	Tourists	Offline	55.30%	Social safety emergency	Risk perception	Behavior
20	David et al., 2024	2024	Spain	Europe	Developed	Risk perception negatively affected the sensation seeking.	No reported	Journal of Safety Research	SEM	471	Drivers	Offline	32.30%	Accidents emergency	Risk perception	Behavior
21	Fung, 2024	2023	China	Asia	Developing	There is a significant positive relationship between risk perception and willingness to search for occupational health and safety information.	No reported	International Journal of Workplace Health Management	HR	486	Flight attendants	Online	15.80%	Accidents emergency	Risk perception	Intention
22	Yang, 2008	2008	China	Asia	Developing	Risk perception has no impact on emergency information seeking behavior.	Uncertainty theory	Qingdao University	SEM	720	Tourists	Online and offline	52.30%	Social safety emergency	Risk perception	Behavior
23	Xu, 2016	2016	China	Asia	Developing	Perceived risk is positively correlated with college students' online information search behavior.	Grounded theory	Anhui University of Finance and Economics	HR	392	College Students	Offline	55.60%	Social safety emergency	Risk perception	Behavior
24	Yin, 2014	2014	China	Asia	Developing	The perceived risk of Internet companion tourists has a positive impact on information search behavior.	No reported	Shandong University	SEM	213	Tourists	Online and offline	55.40%	Social safety emergency	Risk perception	Behavior
25	Huang, 2023	2023	China	Asia	Developing	Risk perception has a negative impact on emergency information seeking behavior.	Grounded theory	Central China Normal University	HR	568	College Students	Online and offline	55.60%	Health emergency	Risk perception	Behavior
26	Lu and Zhang, 2022	2022	China	Asia	Developing	Risk perception has no impact on emergency information seeking behavior.	Attribution theory	Journal of Changchun University	SEM	392	Adults	Online	50.00%	Social safety emergency	Risk perception	Behavior
27	Ma, 2022	2022	China	Asia	Developing	Risk perception has a positive impact on emergency information seeking behavior.	Theory of planned behavior	Guizhou Minzu University	SEM	350	Residents	Offline	49.40%	Health emergency	Risk perception	Behavior
28	Feng, 2022	2022	China	Asia	Developing	Risk perception, health anxiety, behavioral intention, expected performance, social impact and convenience conditions have a significant positive impact on college students' online health information search behavior under the COVID-19.	Theory of planned behavior	Shanxi University of Finance and Economics	SEM	300	College Students	Online and offline	60.00%	Health emergency	Risk perception	Behavior
29	Liu, 2022	2022	China	Asia	Developing	Pearson correlation analysis results indicate a significant positive correlation between consumer risk perception and food safety information search behavior, and risk perception will affect consumer information search behavior to varying degrees.	Theory of planned behavior	Sahnxi Medical University	SEM	1991	Residents	Offline	43.80%	Health emergency	Risk perception	Behavior
30	Dong, 2021	2021	China	Asia	Developing	The study verified that factors such as health information literacy, risk perception, health anxiety, medical convenience, social support, and source characteristics all have a positive impact on the willingness of college students to search for health information during public health emergencies.	Social cognitive theory	Heilongjiang University	HR	283	College Students	Online	50.90%	Health emergency	Risk perception	Behavior
31	Cao et al., 2021	2021	China	Asia	Developing	The influence of users' individual health risk cognition on the variables related to problem solving and the risk information search behavior of COVID-19 and the action path have different differences.	Problem solving situational theory	Journal of Modern Information	HR	292	WeChat users	Online	50.00%	Health emergency	Risk perception	Behavior
32	Wang, 2021	2021	China	Asia	Developing	Research has found that the public's perception of the severity of the epidemic is the highest, followed by perceived susceptibility, and finally risk controllability. The degree of perception of epidemic risk has a significant negative impact on risk information search behavior.	Theory of planned behavior	Zhengzhou University	HR	1015	Residents	Offline	45.80%	Health emergency	Risk perception	Behavior
33	Zheng, 2020	2020	China	Asia	Developing	Risk perception has no impact on emergency information seeking behavior.	Motivation theory	Northeast Agricultural University	HR	387	Supermarket shoppers	Online and offline	51.10%	Social safety emergency	Risk perception	Behavior
34	Liu et al., 2015	2015	China	Asia	Developing	Risk perception significantly positively affects the information search behavior of emergency product consumers.	Theory of planned behavior	Researches in Library Science	HR	476	Residents	Offline	62.00%	Social safety emergency	Risk perception	Behavior
35	Carson et al., 2023	2022	USA	North America	Developed	This study finds information seeking behavior to be the strongest influence on preparedness with other important influences being risk perception, affective response, and intentions to prepare.	Theory of planned behavior	Risk Analysis	SEM	400	Households	Offline	42.40%	Natural disaster emergency	Risk perception	Behavior
36	Kim and Madison, 2020	2020	USA	North America	Developed	Risk perception has a negative impact on emergency information seeking behavior.	Related psychological theory	International Journal of Disaster Risk Reduction	HR	716	Residents	Online	19.20%	Natural disaster emergency	Risk perception	Behavior
37	Ellen and Jan, 2008	2008	Netherlands	Europe	Developed	Results indicate that information needs, risk perception, and current knowledge are direct predictors of intentions to seek information.	Theory of planned behavior	Journal of Risk Research	HR	182	Households	Online	71.00%	Health emergency	Risk perception	Behavior
38	Jiang et al., 2022	2022	China	Asia	Developing	The study found that worry and risk perception lead to negative information-seeking behavior.	Information processing theory	Chinese Journal of Communication	SEM	802	Residents	Online	46.00%	Health emergency	Risk perception	Behavior
39	Liu et al., 2021	2021	China	Asia	Developing	Risk perception has no impact on emergency information seeking behavior.	Theory of planned behavior	Frontiers in Psychology	SEM	1031	Adults	Online	63.50%	Health emergency	Risk perception	Intention
40	Broermann, 2018	2018	Netherlands	Europe	Developed	A regression analysis did not confirm that sensation seeking is a good predictor of risk perception.	Protection motivation theory	Universiteit Twente	HR	124	Adults	Offline	50.00%	Social safety emergency	Risk perception	Behavior
41	Kim et al., 2021	2021	Korea	Asia	Developed	The results revealed positive support that all three variables (risk perception, efficacy beliefs, and subjective norms) enhanced smokers' intentions to seek information.	Protection motivation theory	Business Communication Research and Practice	SEM	311	Smokers	Offline	96.10%	Health emergency	Risk perception	Behavior
42	Huang et al., 2023	2023	China	Asia	Developing	It was found that the Chinese public's risk perception had a positive effect on emergency information seeking behavior.	Theory of planned behavior	INQUIRY	SEM	675	Citizens	Offline	50.20%	Health emergency	Risk perception	Behavior
43	Kellens et al., 2012	2012	Belgium	Europe	Developed	It is shown that risk perception and perceived hazard knowledge are higher for perm anent than tem porary residents, leading to increased information-seeking behavior among the form er group.	Theory of planned behavior	Risk Analysis	SEM	313	Residents	Offline	65.10%	Natural disaster emergency	Risk perception	Behavior
44	Real, 2008	2008	USA	North America	Developed	Risk perception has a negative impact on emergency information seeking behavior.	No reported	Journal of Applied Communication Research	HR	645	Workers	Offline	50.00%	Health emergency	Risk perception	Behavior
45	Yang et al., 2020	2020	USA	North America	Developed	Risk perception has direct significant positive effects on consumers' intention to seek food safety information.	Theory of planned behavior	International Journal of Environmental Researchand Public Health	SEM	774	WeChat users	Online	54.90%	Health emergency	Risk perception	Intention
46	Zeng, 2017	2017	China	Asia	Developing	A major finding of this investigation is that the relationship between risk perceptions and safety behaviors and information-seeking intentions was stronger among those with higher efficacy beliefs than among those with lower efficacy beliefs.	Theory of planned behavior	University of Science and Technology of China	SEM	487	Residents	Offline	59.10%	Accidents emergency	Risk perception	Intention
47	Zhang et al., 2025	2025	China	Asia	Developing	Risk perception has a positive impact on emergency information seeking behavior.	Theory of planned behavior	Journal of Broadcasting and Electronic Media	SEM	300	Residents	Offline	55.00%	Social safety emergency	Risk perception	Behavior
48	Zhang et al., 2025	2025	Korea	Asia	Developed	Risk perception has a positive impact on emergency information seeking behavior.	Theory of planned behavior	Journal of Broadcasting and Electronic Media	SEM	263	Residents	Online	53.00%	Social safety emergency	Risk perception	Behavior
49	Li and Yang, 2025	2025	China	Asia	Developing	Nulliparous women's risk perception has a positive impact on emergency information seeking intention.	Attribution theory	Journal of Applied Communication Research	SEM	424	Nulliparous women	Online	0.00%	Health emergency	Risk perception	Intention
50	Bruwer and Lee, 2025	2025	South Africa	Africa	Developing	The study provides new evidence suggesting a nuanced relationship between risk factors and information seeking.	No reported	International Journal of Hospitality Management	SEM	411	Residents	Offline	40.90%	Health emergency	Psychological risk, Functional risk, Social risk	Behavior

### Meta-analysis results of risk perception and emergency information seeking behavior

Table 2 summarizes the results of the meta-analysis of risk perception and emergency information-seeking behavior. A significant positive correlation was found between overall risk perception and emergency information-seeking behavior (*r* = 0.10, random effects model; *r* = 0.08, fixed effects model; k = 66, *n* = 43,717). The confidence interval did not include zero, indicating that risk perception significantly positively impacted emergency information-seeking behavior, which suggests that risk perception significantly influences emergency information-seeking behavior.

**Table 2:** Meta-analysis results of risk perception and emergency information seeking behavior.

**Variables**	**Model**	***k***	**N**	**Effect size based meta-analysis**				**Heterogeneity**		**Fail-safe N (Rosenthal)**	**Egger's test**	
				***r***	***95% CI***		***Z***	**Q (** ***P*** **-value)**	***I** ^2^*		**t**	***P*** **-value**
Overall	Fixed	66	43,717	0.08	0.07	0.09	16.19	3,292.23^***^	98.03	1,998	0.76	0.46
	Random	66	43,717	0.10	0.04	0.17	2.98	^**^	-	-	-	-
Risk perception-emergency information seeking intention	Fixed	19	14,862	0.04	0.03	0.06	2.52	1,804.35^*^	99.01	780	1.73	0.11
	Random	19	14,862	0.08	0.02	0.14	2.03	^*^	-	-	-	-
Risk perception-emergency information seeking behavior	Fixed	47	28,855	0.11	0.10	0.12	18.04	1,418.23^***^	96.76	4,138	0.81	0.42
	Random	47	28,855	0.12	0.06	0.19	3.66	^***^	-	-	-	-

^*^P < 0.05, ^**^P < 0.01, ^***^P < 0.001.

Specifically, regarding the correlation between risk perception and willingness to search for emergency information, the random effects model indicated a significant positive correlation between the two (confidence interval did not include zero), with a composite effect value of 0.08 (k = 19, *n* = 14862), which was slightly higher than the composite effect value under the fixed effects model (*r* = 0.04).

Concerning the correlation between risk perception and emergency information-seeking behavior, the random effects model indicated a significant positive correlation between the two (confidence interval did not include zero), with a comprehensive effect value of 0.12 (k = 19, *n* = 14,862), which was slightly higher than the comprehensive effect value under the fixed effects model (*r* = 0.11).

### Moderator analysis

As Hunter and Schmidt (2004) highlighted, differences in research results may be attributable to statistical artifacts or potential regulatory factors, such as disaster type and demographic and methodological characteristics. Therefore, in addition to core hypothesis testing, we conducted an exploratory moderation factor analysis to evaluate whether our findings differed owing to the demographic and methodological features primarily studied in the database. Table 3 presents the results of the analysis wherein we tested two demographic characteristics, national development level (developed and developing countries) and male proportion (male proportion greater than or equal to 50% and male proportion less than 50%), as moderating factors for risk perception and the willingness to search for emergency information. Additionally, we considered emergency types [i.e., four types of disaster emergencies (natural disaster emergency, accident emergency, health emergency, and social security emergency)] and several methodological features [i.e., publication time, sample collection strategy (online or offline), and measurement methods (SEM and HR) as moderating variables]. Overall, publication time had a weak or no moderating effect (e.g., in the relationship between overall risk perception and overall emergency information-seeking behavior, risk perception and emergency information-seeking willingness, and risk perception and emergency information-seeking behavior). The regression analysis of publication time showed no impact (the estimated value was not significant). In the analysis of cultural background (national development level), developed countries had a slightly stronger moderating effect on the relationship between overall risk perception and overall emergency information-seeking behavior, as well as on the relationship between risk perception and emergency information-seeking intention, than developing countries. Notably, developing countries had a stronger moderating effect on the relationship between risk perception and emergency information-seeking intention than developed countries (0.12 vs. 0.07). The four types of emergencies had different regulatory effects. In the relationship between total risk perception and total emergency information-seeking behavior, health and natural disaster emergencies had a significant positive regulatory effect, whereas accidents and social security emergencies had a weak negative regulatory effect (−0.06 and −0.02, respectively). Noteworthily, in the relationship between risk perception and emergency information-seeking willingness, health, accident, and natural disaster emergencies had a significant positive regulatory effect, while social security emergencies had a significant negative regulatory effect. In the relationship between risk perception and emergency information-seeking behavior, health, natural disaster, and social security emergencies had a significant positive regulatory effect, whereas accident emergencies had a significant negative regulatory effect. Concerning the method of collecting sample population data, offline methods, such as field surveys, had a slightly stronger moderating effect on all relationships than online methods, such as email, phone, and online questionnaires. Regarding measurement method, the significant positive regulatory effect of SEM measurement was stronger than that of HR measurement. Concerning sample population characteristics, the significant positive regulatory effect of a male proportion greater than 50% (i.e., male-dominated) on all relationships was stronger than that of a male proportion less than 50% (i.e., female-dominated).

**Table 3:** Results of moderators' analysis.

**Relationships**	**Moderators**	**Model**	***k***	***N***	**Estimate**	**95%CI**		**Z**	**Q within**	**Q between**	**Q (P-value)**
Overall risk perception-overall emergency information seeking intention	Publication year	Regression	66	43,717	0.00	−0.01	0.02	0.58	-	-	0.34
	National development level-developed	Fixed	21	11,630	0.10	0.08	0.12	10.87	3,283.87	8.36	301.70^***^
	National development level-developing	Fixed	45	32,087	0.07	0.06	0.08	12.35			2,982.17^***^
	Measure method-HR	Fixed	15	6,594	0.03	0.01	0.05	2.41	3,274.25	17.98	356.18^*^
	Measure method-SEM	Fixed	51	37,123	0.09	0.08	0.10	16.56			2,918.07^***^
	Colleted type-offline	Fixed	21	11,410	0.12	0.10	0.14	12.70	3,260.98	31.24	784.83^***^
	Colleted type-online	Fixed	35	27,444	0.07	0.06	0.08	11.17			2,325.34^***^
	Colleted type-mixed	Fixed	10	4,863	0.04	0.01	0.07	2.71			150.82^***^
	Gender-male% < 50%	Fixed	31	24,587	0.02	0.01	0.04	3.74	3,125.72	166.51	2,199.08^***^
	Gender-male%>50%	Fixed	35	19,130	0.15	0.13	0.16	20.36			926.64^***^
	Emergency type-accidents emergency	Fixed	2	1,444	−0.06	−0.13	−0.01	−2.04	3,130.38	161.84	76.49^*^
	Emergency type-health emergency	Fixed	35	27,643	0.12	0.11	0.13	19.22			2,565.86^***^
	Emergency type-natural disaster emergency	Fixed	9	6,473	0.08	0.06	0.11	6.73			65.59^***^
	Emergency type-social safety emergency	Fixed	20	9,601	−0.02	−0.04	−0.01	−2.29			422.45^*^
Risk perception-emergency information seeking intention	Publication year	Regression	19	14,862	0.08	0.03	0.12	3.26	-	-	0.11
	National development level-developed	Fixed	5	3,468	0.17	0.13	0.20	10.49	1,687.98	116.37	22.99^***^
	National development level-developing	Fixed	14	11,394	−0.04	−0.05	−0.02	−3.55			1,665.99^***^
	Measure method-HR	Fixed	3	1,952	0.21	0.12	0.29	4.68	1,785.89	18.44	0.00^***^
	Measure method-SEM	Fixed	16	12,910	0.02	0.00	0.03	1.69			1,785.89
	Colleted type-offline	Fixed	-	-	-	-	-		1,804.35	0.00	-
	Colleted type-online	Fixed	19	14,862	0.02	0.01	0.04	2.52			1,804.35^***^
	Colleted type-mixed	Fixed	-	-	-	-	-	-			-
	Gender-male% < 50%	Fixed	14	12,380	0.03	0.02	0.04	0.26	1,789.95	14.41	1,789.95^*^
	Gender-male%>50%	Fixed	5	2,482	0.08	0.04	0.11	4.55			29.95^***^
	Emergency type-accidents emergency	Fixed	1	487	0.21	0.12	0.29	4.69	1,546.74	257.61	0.00^***^
	Emergency type-health emergency	Fixed	11	8,992	0.11	0.09	0.13	9.56			1,515.28^***^
	Emergency type-natural disaster emergency	Fixed	2	1,420	0.09	0.04	0.14	3.44			2.19^***^
	Emergency type-social safety emergency	Fixed	5	4,450	−0.18	−0.20	−0.15	−11.78			29.28^***^
Risk perception-emergency information seeking behavior	Publication year	Regression	47	28,855	0.00	−0.01	0.02	0.58	-	-	0.56
	National development level-Developed	Fixed	16	6,585	0.07	0.04	0.09	5.81	1,402.01	16.22	252.92^***^
	National development level-developing	Fixed	31	22,270	0.12	0.11	0.13	17.55			1,149.09^***^
	Measure method-HR	Fixed	14	4,364	0.02	0.01	0.04	1.18	1,354.18	64.05	338.65^*^
	Measure method-SEM	Fixed	33	24,491	0.13	0.12	0.14	19.71			1,015.53^***^
	Colleted type-offline	Fixed	21	6,373	0.12	0.10	0.14	12.71	1,391.73	26.49	784.83^***^
	Colleted type-online	Fixed	16	19,932	0.11	0.10	0.14	13.54			456.08^***^
	Colleted type-mixed	Fixed	10	2,550	0.04	0.01	0.07	2.72			150.82^**^
	Gender-male% < 50%	Fixed	17	17,364	0.04	0.02	0.06	4.76	1,302.11	116.11	430.43^***^
	Gender-male%>50%	Fixed	30	11,491	0.17	0.15	0.18	20.47			871.68^****^
	Emergency type-accidents emergency	Fixed	1	471	−0.34	−0.42	−0.26	−7.66	1,310.07	108.16	0.00^***^
	Emergency type-health emergency	Fixed	24	13,976	0.12	0.11	0.14	16.72			1,048.92^***^
	Emergency type-natural disaster emergency	Fixed	7	6,473	0.08	0.05	0.11	5.79			63.30^***^
	Emergency type-social safety emergency	Fixed	15	7,935	0.11	0.08	0.14	7.85			197.85^***^

^*^P < 0.05, ^**^P < 0.01, ^***^P < 0.001, ^****^P < 0.0001.

### Analysis of publication bias

**Figure 3:**
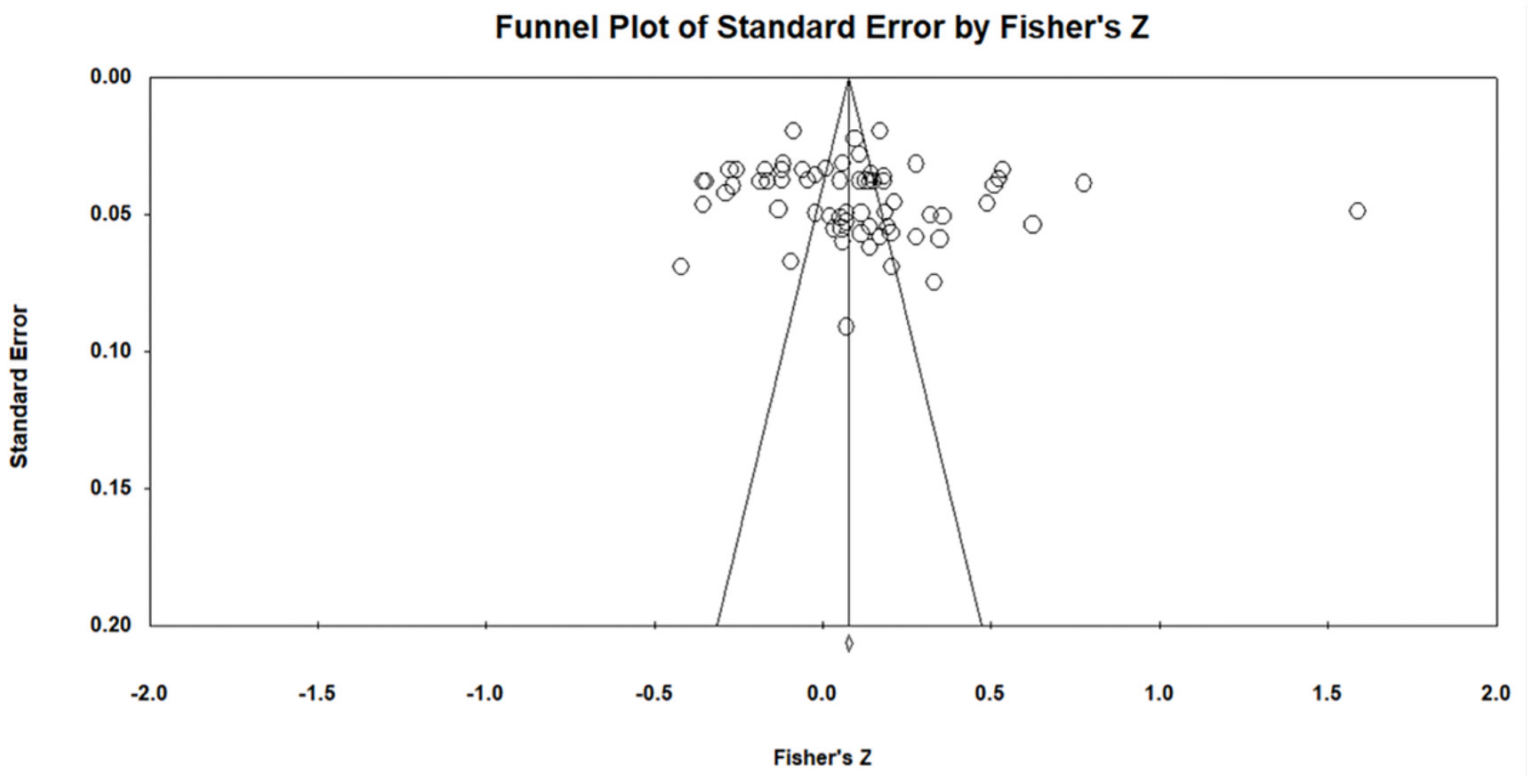
Funnel plot of all included articles.

Publication bias (i.e., selective reporting) refers to any situation during the processes of data collection, analysis, interpretation, and publication that may result in a systematic deviation of the conclusions from true results, such as self-examination by authors to meet theoretical expectations or the tendency of journals to support significant results. To alleviate this concern, we employed Failsafe N (Rosenthal) and Egger regression analyses to test for publication bias, both of which are usually based on defining the distribution of the effect size for a given zero-order relationship. Table 1 presents specific information on the publication bias analysis of the included studies. Owing to the inclusion of studies that did not satisfy the conditions for two or more articles and the fact that the standard error was not zero, the analysis of publication bias was limited, and the results could not be obtained. Table 1 presents the results of the publication bias analysis. The Failsafe N (Rosenthal) value was significantly greater than 5K + 10 (where K represents the number of included studies), the intercept terms of the Egger regression analysis were mostly equal to or close to zero, and all *p*-values were non-significant. For example, for overall risk perception and emergency information-seeking intention, the Failsafe N was 1,998 and 780, respectively (significantly greater than 5K + 10, where K is 66 and 19, respectively), and the p-values were 0.46 and 0.11, respectively (both greater than or equal to 0.05). Figure 3 depicts their overall funnel plot, which indicates that the effect values of all included studies were almost uniformly distributed. The funnel plot was utilized to check whether there was the possibility of reporting bias in the study. Therefore, we can conclude that, although the current study exhibited publication bias, its impact on the results was negligible.

### Article quality evaluation results

Table 4 presents the risk of bias assessment are presented in Table 4. The bias tools primarily included five aspects: recall description, blind methods, random generation, task concealment, and other biases. Two reviewers conducted the biased risk of bias. Overall, the low, high, and unknown risk rates were 80.4%, 11.3%, and 8.3%, respectively. The average score of the 42 articles was low (80.4%), while five articles had high scores (11.3%), and three articles had unclear ratings (8.3%).

**Table 4:** Risk-of-bias assessment of eligible articles.

**Risk-of-bias criteria**	**Description**	**Risk-*n* (%)**		
		**High**	**Low**	**Unclear**
Selection bias	Is there a random allocation of subjects in the experiment? Is there random sampling in the study?	1 (2.0)	46 (92.0)	3(6.0)
Performance bias	Are the conditions outside the experiment consistent? Do investigators and respondents know the purpose of the study?	6 (12.0)	42(84.0)	2 (4.0)
Detection bias	Is the standard measurement tool the same for the experimental group and the control group?	3(6.0)	42 (84.0)	5 (10.0)
Attrition bias	What is the proportion of valid questionnaires, is there a missing value in the answers, and are there explanations for the above?	6(12.0)	41 (82.0)	3(6.0)
Reporting bias	Are the results of the study reported truthfully (i.e., both positive and negative results are reported)?	12 (24.0)	31(62.0)	7 (14.0)
Other bias	Is the basic information description of the sample complete, and are the reliability and validity of the measurement tool in the study reported?	6 (12.0)	39(78.0)	5 (10.0)

## Discussion

To the best of our knowledge, this is the first study to examine risk perception's impact on emergency information-seeking behavior using a meta-analysis. It systematically integrated data from 50 relevant studies and revealed a significant positive correlation between risk perception and emergency information-seeking behavior. This discovery not only offers theoretical support for information dissemination strategies in emergency situations but also provides practical guidance for enhancing public emergency response capabilities. Our comprehensive analysis found that various factors—such as disaster type, individual differences, and information dissemination channels—influence the relationship between risk perception and emergency information-seeking behavior. Subsequently, we discuss the mechanisms underlying these factors and their significance to the research results.

The meta-analysis found a significant positive correlation between risk perception and emergency information-seeking behavior [effect value of 0.10, 95% confidence interval (0.04, 0.17)], indicating that an improvement in risk perception significantly promotes individuals' emergency information-seeking behavior. Although 0.1 is a small effect, small effect values are common in cross-situational meta-analyses involving human behavior. This is because individual behavior is influenced by multiple cognitive situational factors, and the independent effect of a single predictor variable is often diluted by other moderating variables. This study's total sample size was 29,014 people, and the large sample size makes the small effect values significant in practice. According to the “effect value population influence” model (Rosenthal, 1990), an effect value of *r* = 0.10 can be converted into behavior changes in about 10,000 people out of 1 million (i.e., 1% of the population actively seeks emergency information due to increased risk perception). During public health events such as the COVID-19 pandemic or large-scale natural disasters, this proportion of behavioral changes significantly reduces the group infection or casualty rate, reflecting the cumulative value of “small effects, big impacts” (Cohen, 1988; Li et al., 2020; Rosenthal, 1990; Tang and Li, 2025). This discovery aligns with theories such as the TPB and RISP, which suggest that when an individual perceives an increase in risk, it triggers their need and willingness to search for relevant information to reduce uncertainty and help them take appropriate response measures (Kogler et al., 2022; Li et al., 2020; Mokry, 2019; Wen, 2019; Zhao and Cai, 2009).

Overall, the results revealed that risk perception positively impacts emergency information-seeking behavior. One possible reason is that an increase in risk perception makes individuals aware of the existence of potential threats, thereby stimulating their willingness to actively search for emergency information. This information-seeking behavior not only provides individuals with a more comprehensive understanding of the nature, potential impacts, and coping strategies of risks but also enhances their sense of risk control, thereby improving their confidence and ability to cope with risks. For example, during public health events, the higher an individual's perception of disease risk, the more likely they are to actively search for relevant health information, prevention and control measures, and vaccination information to better protect their own and their family's health (Liao et al., 2018; Zhao and Cai, 2009; Tang and Li, 2025).

Additional contextual analysis revealed significant differences in the impact of risk perception on emergency information-seeking behavior in different types of disaster scenarios. Among them, the driving effect of risk perception on information-seeking behavior was most significant in health emergency scenarios, with a stronger impact than in other scenarios, such as natural disasters (effect value of 0.12). The results suggested that the effect value in natural disaster scenarios was 0.08, whereas in health emergency scenarios, because of the concealment of disease transmission, directness of health threats, and professionalism of prevention and control measures, the correlation between individual risk perception and information-seeking behavior was stronger. This difference may be attributable to the unique biological infectivity, latency uncertainty, and social panic amplification effects of health security events, which result in public demands for information that is immediate and accurate. By contrast, although the visible destructive nature of natural disasters triggers higher risk perception, the “intangible threat” of pathogen transmission, the professional threshold of prevention and control knowledge, and the urgency of direct association with individual health in health emergency events are more likely to stimulate sustained information-seeking behavior to alleviate cognitive uncertainty and decision anxiety (Hovick et al., 2014; Kellens et al., 2012; Liao et al., 2018; Zeng et al., 2024; Zhao and Cai, 2009).

By contrast, other types of emergencies, such as accidents and social security emergencies—despite also having an impact on individuals' risk perception—had a relatively small promoting effect on information-seeking behavior owing to differences in their occurrence mechanism, scope of impact, and individual response methods compared to natural disasters. For example, accidents and disasters are usually sudden, and individuals may only realize the existence of risk after the event occurs. At this time, their information-seeking behavior is more influenced by the urgency of the event and the on-site environment, which may involve complex social and human factors. Individuals' risk perception is not only influenced by the threat of the event itself but also by various factors such as public opinion and group emotions, which may affect their motivation and method of information-seeking behavior (Chao et al., 2016; David et al., 2024; Fung, 2024; Kaur, 2018; Piracha, 2021). The impact of social security emergencies is minimal, possibly because the risk perception of such events often manifests in a delayed manner. For example, delays in pension payments can only be detected the following month, making it difficult to trigger immediate searches. Furthermore, they only affect specific groups, such as certain insured individuals, leading to weak perception diffusion and a small search group base. Moreover, they involve complex policy terms, rendering it difficult for most people to identify the causes of risks, and thus, their searches lack specificity. Additionally, people are accustomed to relying on the government to solve social security issues, assuming that the government will proactively inform them, resulting in a low willingness to actively search. Furthermore, significant regional differences exist in social security policies, and they are often updated; thus, the information retrieved is often invalid, further weakening search motivation. Even if they perceive risks, most people merely ask relatives and friends about it or wait for notification, and their proactive search behavior is far lower than after accidents or natural disasters (Ajzen, 1991; Kellens et al., 2012; Slovic, 1987; Zeng et al., 2024).

Noteworthily, the negative moderating effects of accident emergency response (such as industrial accidents) and social security emergency response (such as terrorist attacks) were −0.06 and −0.02, respectively. The technical complexity of accident emergency response may result in information overload for the public, or a decrease in trust in information owing to disputes over the attribution of responsibility. Social security emergencies may lead individuals to develop a perception that information acquisition is not helpful for risk response owing to information control or disorder and, thus, adopt avoidance strategies. We observe that accident emergency response exhibited a positive regulation at the level of “risk perception and willingness to search for emergency information,” but turned negative at the level of actual behavior. This contradiction reflects the “willingness behavior gap,” where individuals are aware of the importance of information but are limited by acquisition costs (such as environmental safety and information credibility) and fail to translate it into action, highlighting the critical impact of situational constraints on behavioral decision-making (Ajzen, 1991; Chao et al., 2016; David et al., 2024; Kaur, 2018; Piracha, 2021; Slovic, 1987).

The significant differentiation effect of emergency types highlights the role of risk attributes in shaping information behavior: Health and natural disaster emergencies—as high threat and high uncertainty scenarios—had a significant positive moderating effect on the relationship between risk perception and emergency information seeking. Their core driving logic lies in the difference between the “visibility” and “professionalism” of risks. Health emergency events force the public to reduce cognitive uncertainty through high-frequency information searches due to the concealment of pathogen transmission, the immediacy of health threats, and the technical threshold of prevention and control knowledge. Although natural disasters such as earthquakes and floods have visible destructive effects, their response strategies (such as evacuation routes and material reserves) have clear and urgent information needs, and risk perception is directly transformed into goal-oriented seeking behavior (Ajzen, 1991; Kellens et al., 2012; Slovic, 1987).

Across diverse cultural contexts, the moderating effect of the national development level exhibited subtle differences. The results revealed that developed countries had a slightly stronger moderating effect on the relationship between risk perception and information-seeking willingness/behavior than developing countries, which may be associated with the mature emergency information infrastructure of the former (e.g., intelligent warning systems and public health information platforms). The public can obtain authoritative information in real time through multiple channels, and risk perception is more easily translated into precise actions. However, developing countries had a more significant moderating effect (0.12 vs. 0.07) on risk perception's direct impact on information-seeking behavior. They may be attributable to the limited information dissemination channels, which implies that the public needs to rely on active searches to fill the information gap, thus strengthening the driving role of risk perception on behavior. The adjustment results for the gender ratio indicated that in groups with a male proportion of ≥ 50%, risk perception had a stronger positive impact on information-seeking behavior, which may be related to gender role socialization. Men are frequently assigned the role of “problem solvers” and tend to formulate strategies through information acquisition when facing risks. Meanwhile, women may rely more on social support networks; hence, their information-seeking behavior is more deeply influenced by emotions or advice from others (Kim et al., 2021; Li and Yang, 2025).

More nuanced methodological analyses also provided us with valuable insights. The moderating effect of sample collection and measurement methods provides empirical evidence for future research designs: offline field surveys had a stronger moderating effect on relationships than online data collection, reflecting that the situational authenticity of offline samples is closer to the behavioral logic of emergency scenarios, and they avoid the “social approval bias” of online environments. The positive moderating effect of SEM was superior to HR, reflecting SEM's advantage in handling multidimensional latent variable relationships. Further, SEM can more accurately capture the indirect impact of risk perception on information-seeking behavior through mediating variables such as anxiety and coping efficacy. Notably, publication date did not exhibit a significant moderating effect, indicating that the core correlation between risk perception and information-seeking behavior has cross-temporal stability and has not undergone fundamental changes with the advancement of emergency management technology (Li et al., 2020; Tang and Li, 2025).

### Theoretical and practical implications

This study breaks through the universality assumption of traditional risk perception theory and confirms “situational sensitivity” in relation to emergency information-seeking behavior, adding a typological analysis dimension to emergency communication theory. At a practical level, emergency management departments need to develop differentiated strategies for different types of disasters. For health emergencies and natural disasters, real-time and accurate information should be strengthened (such as short science videos and intelligent terminal warnings), and addressing information transparency and trust rebuilding (such as third-party source intervention and multi-party communication mechanisms) should be prioritized. In response to the active search needs of the public in developing countries, efforts should be invested to strengthen the construction of localized emergency information platforms; to address gender differences, more targeted communication channels should be designed (e.g., female-dominated social media accounts focusing on mental health and family protection information) (Ajzen, 1991; Batrancea et al., 2025; Kellens et al., 2012; Slovic, 1987).

This study's results carry key practical implications for improving the effectiveness of information dissemination and public emergency response capabilities. First, understanding the impact mechanism of risk perception on emergency information-seeking behavior can help government departments and relevant institutions release and disseminate risk information more specifically when working on emergency management. Increasing the public's risk perception level can stimulate their willingness to actively search for emergency information and enhance their attention to and demand for emergency information, thus improving information dissemination's effectiveness (Batrancea et al., 2022a,b; Kellens et al., 2012; Wen, 2019).

Second, this study revealed differences in the relationship between risk perception and information-seeking behavior in different types of disasters, suggesting that relevant departments should adopt different information dissemination strategies when dealing with different types of disasters. For example, to prevent and respond to natural disasters, various information dissemination channels should be utilized, accurate and timely warnings should be released in advance, public risk awareness education should be strengthened, and risk perception and emergency information-seeking capabilities for natural disasters should be improved. In public health incidents, it is necessary to pay greater attention to the transparency and credibility of information, the timely release of epidemic dynamics, prevention and control measures, and scientific knowledge to alleviate public panic, guide people to search for and use health information reasonably, and actively cooperate with epidemic prevention and control efforts (Shen et al., 2022; Slovic, 1987).

Additionally, focusing on individual differences and the moderating effect of information dissemination channels can help further optimize the effectiveness of emergency information dissemination. More targeted information content and dissemination methods should be designed and provided to groups with different individual characteristics, such as age, educational background, and gender. For example, for the younger generation, social media and other online platforms can be utilized to publish emergency information in various forms that are easy to understand and disseminate, and traditional media channels and community promotions can be used to ensure that they obtain important emergency information in a timely manner. Simultaneously, it is necessary to strengthen collaborative cooperation between traditional and social media, leveraging their respective advantages; improve the quality and coverage of emergency information dissemination; better satisfy the information needs of the public in emergency situations; and enhance people's emergency response capabilities (Li and Yang, 2025; Slovic, 1987).

This study systematically explored risk perception's impact on emergency information-seeking behavior and its moderating factors through meta-analysis. This approach offers a comprehensive perspective and theoretical support for further understanding this relationship. In practical applications, the mechanism of risk perception and moderating factors—such as disaster type, individual differences, and information dissemination channels—should be fully considered to develop more scientific and effective emergency information dissemination strategies to improve the public's ability to respond, ensuring safety in emergency situations.

### Limitations and future development directions

Although this study makes valuable contributions, some limitations persist. First, the literature reviewed was concentrated in certain countries and regions and cannot fully represent risk perception and emergency information-seeking behavior worldwide. Significant differences may exist in people's risk perceptions, information search habits, and responses to disasters in different cultural backgrounds, and the geographical limitations of existing research affect the generalizability of the results (Li et al., 2020).

Second, the literature contains diverse measurement tools and indicators for evaluating risk perception and emergency information-seeking behavior, resulting in heterogeneous results. Some studies utilized subjective perception measurement methods, whereas others concentrated on objective behavioral recording. Differences in measurement methods complicate cross-study comparisons and make integration complex (Barends and Rousseau, 2018; Hedges and Tipton, 2009).

Finally, this study has some limitations in the research design. Most included studies had a cross-sectional design, rendering it difficult to determine the causal relationship between risk perception and emergency information-seeking behavior. Cross-sectional studies can only reflect associations at specific time points and cannot capture the dynamic changes and causal directions of variables over time, nor can they provide in-depth explanations of the mechanisms underlying the moderating variables (Barends and Rousseau, 2018).

Future research should focus on the following directions: First, samples should be expanded to cover more countries, regions, and cultural groups to enhance the universality and cross-cultural applicability of the results. By comparing risk perception and emergency information-seeking behavior across different cultural backgrounds, we can gain a deeper understanding of the role of cultural factors and provide a foundation for developing cross-cultural emergency information dissemination strategies. Second, developing and validating standardized risk perception and emergency information-seeking behavior measurement tools will improve the consistency and comparability of results, reduce heterogeneity caused by differences in measurement methods, and enable more accurate comparisons between different studies and integration. Third, longitudinal research designs should be strengthened. A longitudinal research design should be utilized to track the changes in risk perception and emergency information-seeking behavior of the same group at different time points, which will help determine causal relationships and temporal evolution patterns, and increase the understanding of the dynamic mechanisms of the moderating variables.

Fourth, the mechanisms of moderating variables should be further explored. Moderating variables that impact the relationship between risk perception and emergency information-seeking behavior include individual differences, disaster types, and information dissemination channels. To explore the interaction between individual differences and specific types of disasters, it is important to examine the specific effects of information dissemination channels in different types of disasters and cultural backgrounds, and their interactions with risk perceptions. Additionally, attention should be paid to other potential moderating variables, such as social support and community environment, to more comprehensively elucidate the complex relationship between risk perception and emergency information-seeking behavior.

Fifth, multidisciplinary perspectives should be integrated. We need to encourage interdisciplinary collaborative research and explore risk perception and emergency information-seeking behavior from multiple perspectives by combining theories and methods from psychology, sociology, communication studies, information technology, and other disciplines. Psychological experimental methods and models can be employed to accurately measure the psychological mechanisms of risk perception, drawing on the theoretical framework of sociology to study the role of social networks and social capital. Communication studies can be utilized to study and evaluate the impact of media channels and information presentation methods, and we can explore the potential application of emerging technologies in emergency information dissemination and risk perception monitoring, considering the development trends of information technology (Li et al., 2020).

## Conclusion

Risk perception significantly impacts how individuals assess risk, make decisions, and behave. While numerous studies have examined risk perception's impact on emergency information-seeking behavior, the association remains unclear. This study established a theoretical framework and analyzed risk perception's impact on emergency information-seeking behavior using meta-analysis. Fifty relevant studies (29,014 participants) covering risk perception and information-seeking behavior in four emergency scenarios were included. The results revealed a significant positive correlation between risk perception and emergency information-seeking behavior. Additional exploratory analysis indicated different impacts of risk perception on information-seeking behavior in each type of emergency. Health and natural disaster emergencies had a significant positive moderating effect, whereas accidents and social security emergencies had a significant negative moderating effect. We found significant differences in the moderating effects of demographics (national development level and male proportion) and methodology (publication time, sample collection strategy, and measurement method). Finally, we evaluated publication bias and literature quality to determine the robustness and scalability of the results. To the best of our knowledge, this is the first meta-analysis study on risk perception and emergency information-seeking behavior. It summarizes rich empirical knowledge on this relationship. This study followed contemporary meta-analysis guidelines and best practices to generate transparent and replicable scientific findings. Our findings can help improve information dissemination's effectiveness in emergency situations and offer a theoretical foundation for strengthening public emergency response capabilities.

## Data Availability

The original contributions presented in the study are included in the article/supplementary material, further inquiries can be directed to the corresponding authors.
